# Chinese species of genus *Notopygus* Holmgren (Hymenoptera, Ichneumonidae, Ctenopelmatinae) with description of a new species

**DOI:** 10.3897/zookeys.387.6671

**Published:** 2014-03-10

**Authors:** Shu-Ping Sun, Mao-Ling Sheng

**Affiliations:** 1 General Station of Forest Pest Management, State Forestry Administration, Shenyang, Liaoning, 110034, China

**Keywords:** Ctenopelmatini, *Notopygus*, new species, key, host, Pamphiliidae, *Neurotoma sibirica*, China

## Abstract

A new species, *Notopygus longiventris* Sun & Sheng, **sp. n.**, collected from Benxi County, Liaoning Province, China, and *N. emarginatus* Holmgren, 1857, reared from *Neurotoma sibirica* Gussakovskij (Hymenoptera, Pamphiliidae) from Haicheng, Liaoning Province, are reported. The new species is placed within existing key to species.

## Introduction

*Notopygus* Holmgren, 1857, belonging to the tribe Ctenopelmatini of the subfamily Ctenopelmatinae (Hymenoptera: Ichneumonidae), comprises 16 species (Yu et al. 2012), of which one is from the Oriental Region, nine from the Western Palaearctic (five of them also found in the Eastern Palaearctic), six from the Eastern Palaearctic and four are from the Nearctic. The Palaearctic species were revised by Kasparyan (2002). A key to the species of the Eastern Palaearctic Region was given by Kasparyan and Khalaim (2007). The status of the genus was elucidated by Townes (1970) and by Kasparyan (2002).

Two species have been known from China, of which one, *Notopygus emarginatus* Holmgren, 1857, found in Liaoning, was previously mistaken for *Notopygus insignis* Kriechbaumer, 1891 (Chen et al. 2007). *Notopygus raricolor* (Aubert 1985), found in Sichuan, was originally described only from the male, and was redescribed, and its systematic position discussed by Kasparyan (2002).

Ecological and morphological notes on *Notopygus bicarinatus* Teunissen, 1953 (= *Notopygus minkii* Vollenhoven, 1878) were made by Holuša et al. (2011). The biology of *Notopygus emarginatus* Holmgren, 1857 in Haicheng, Liaoning Province (as *Notopygus insignis*) was described by Chen et al. (2007).

In this article, three species of *Notopygus* are reported, of which one was reared from *Neurotoma sibirica* Gussakovskij (Hymenoptera, Pamphiliidae) in Haicheng, Liaoning Province, and one collected from Benxi, Liaoning Province, is new to science.

## Materials and methods

The unique specimen of *Notopygus longiventris* Sun & Sheng, sp. n. was collected by intercept trap (Li et al. 2012) in the forests of Benxi County, Liaoning Province (China). Specimens of *Notopygus emarginatus* were reared from *Neurotoma sibirica* Gussakovskij (Hymenoptera, Pamphiliidae) in Haicheng, Liaoning province, and collected while they were ovipositing into their hosts (Figure 11), as well as being collected with intercept traps and hand nets in the forests of Benxi and Kuandian, Liaoning province. The forest of Benxi is composed of mixed deciduous angiosperms and evergreen conifers, mainly comprising *Pinus koraiensis* Sieb. & Zucc., *Acer mono* Maxim., *Juglans mandshurica* Maxim., *Prunus padus* L., *Fraxinus rhynchophylla* Hance and *Ulmus pumila* L. The forest of Kuandian is composed of mixed deciduous angiosperms, mainly comprising *Quercus wutaishanica* Blume, *Celtis bungeana* Bl., *Larix gmelinii* (Rupr.) Rupr., *Prunus padus* and *Fraxinus rhynchophylla*.

Images of whole insects were taken with a Canon Power Shot A650 IS. Other images were taken using a Cool Snap 3CCD attached to a Zeiss Discovery V8 Stereomicroscope and captured with QCapture Pro version 5.1.

Specimens were compared with material from the Natural History Museum (NHM), London, UK and the Zoologische Staatssammlung München (ZSM), Germany.

The type specimen is deposited in the Insect Museum, General Station of Forest Pest Management (GSFPM), State Forestry Administration, People’s Republic of China.

## Results

### Notopygus

Holmgren, 1857

http://species-id.net/wiki/Notopygus

Notopygus Holmgren, 1857. Kongliga Svenska Vetenskapsakademiens Handlingar, 1(1)(1855): 115. Type-species: *Notopygus emarginatus* Holmgren. Designated by Viereck 1912.

#### Diagnosis.

Mandibular teeth of equal length, or lower tooth slightly longer than upper tooth. Tarsal claws not pectinate or weakly pectinate at base. Lateral longitudinal carina present. Apical portion of metasoma almost cylindric or depressed. First tergite without glymma. Posterior margin of tergite 8 rather frequently strongly projecting upwards. Female hypopygium strongly enlarged, widely convex at posterior margin. Ovipositor sheaths short, harddly projecting beyond metasoma apex, compressed. Ovipositor up-curved.

#### In Kasparyan’s (2002) key to species, the new species can be inserted as follows:

**Table:** 

7 (6)	Tergite III almost entirely red, matte, finely shagreened. Hind tibia as long as 1st–3rd segments of hind tarsus combined. Posterior margins of tergites IV–VI widely emarginate, usually coriaceous, whitish.
7a (7b)	Posterior margin of tergite 6 truncate (female). Posterior margin of tergite 8 weakly projecting upwards. Fore wing with vein 1cu-a distal to 1/M. Hind tibia distinctly shorter than 1st–3rd segments of hind tarsus combined	*Notopygus longiventris* Sun & Sheng, sp. n.
7b (7a)	Posterior margin of tergite 6 strongly concave forwardly (female). Posterior margin of tergite 8 sharply up-curved and projecting upwards. Fore wing with vein 1cu-a opposite or slightly basal of 1/M. Hind tibia as long as 1st–3rd segments of hind tarsus combined	*Notopygus emarginatus* Holmgren

### Notopygus longiventris

Sun & Sheng sp. n.

http://zoobank.org/2DE64C1A-8290-4F58-90C2-2FF1A5CABD60

http://species-id.net/wiki/Notopygus_longiventris

Figures 1–10

#### Etymology.

The specific name is derived from the elongate body.

#### Type.

Holotype, female, CHINA: Benxi County, Liaoning Province, 4 July 2013, collected with intercept trap (Tao Li).

#### Diagnosis.

Malar space very narrow, approximately 0.17 times as long as basal width of mandible. Frons strongly divided into two half, lower half deeply concave. Lower end of occipital carina joining hypostomal carina at base of mandible. Fore wing with vein 1cu-a distal to 1-M. Posterior margin of tergite 6 truncate. Posterior margin of tergite 8 slightly projecting upwards. Tergites 2, 3 and basal portion of tergite 4, lateral portions of tergites 5 and 6 brownish red.

#### Description.

Female. Body length 15.0 mm. Fore wing length 9.5 mm. Antenna length 10.5 mm.

**Head.** Face (Fig. 2) 1.7 times as wide as long, median portion slightly convex longitudinally; with uneven punctures and longitudinal wrinkles; upper margin with a small median tubercle; between antenna socket and eye with obvious longitudinal concavity. Clypeus 3.3 times as wide as long, with shallow uneven punctures, median section of apical margin thick. Mandible (Fig. 2) distinctly elongate, with dense longitudinal wrinkles and fine punctures; upper tooth slightly shorter than lower tooth. Subocular sulcus absent. Malar space 0.17 times as long as basal width of mandible. Gena in dorsal view slightly longer than length of eye, with dense fine punctures and a few large uneven punctures. Vertex (Fig. 3) with dense indistinct punctures, postero-median portion distinctly concave. Postocellar line as long as ocular-ocellar line. Dorsal half of frons flat, with indistinct, weak, irregular wrinkles; lower half deeply concave, smooth, shiny. Antenna with 40 flagellomeres; ratios of lengths from first to fifth flagellomeres: 2.3:2.2:2.0:1.8:1.7. Occipital carina complete, lower end joining hypostomal carina at base of mandible.

**Figures 1–10.**
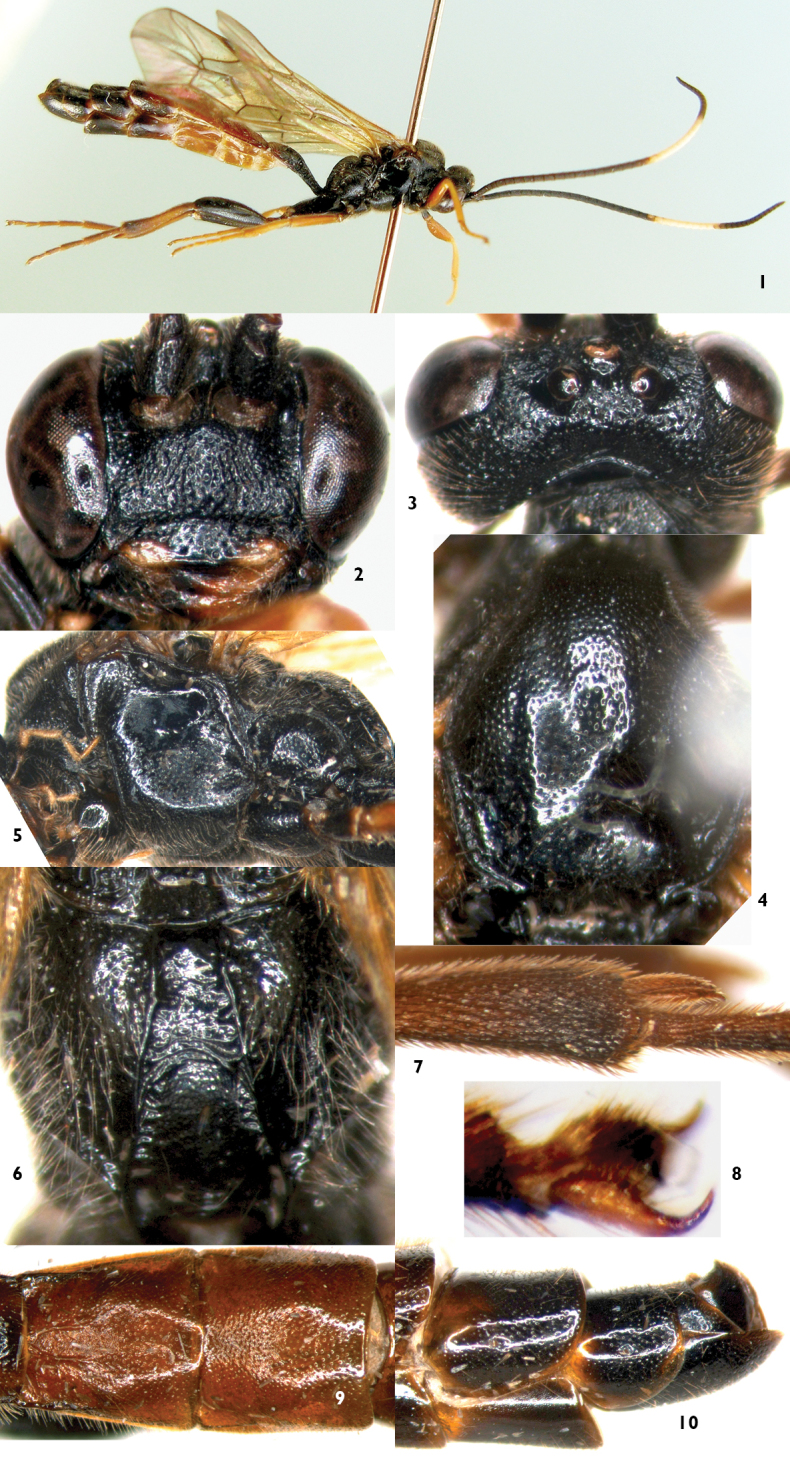
*Notopygus longiventris* Sun & Sheng, sp. n. Holotype. Female **1** Habitus, lateral view **2** Head, anterior view **3** Head, dorsal view **4** Mesoscutum **5** Mesopleuron **6** Propodeum **7** Inner apical portion of hind tibia **8** Hind claw **9** Tergites 2–3 **10** Apical portion of metasoma, lateral view.

**Mesosoma.** Anterior margin of pronotum with fine, blurry longitudinal wrinkles; lateromedian portion with dense oblique transverse wrinkles; upper posterior portion with distinct punctures. Epomia distinct. Mesoscutum (Fig. 4) smooth, shiny, with fine, uneven punctures. Scuto-scutellar groove wide, with short longitudinal wrinkles. Scutellum with dense punctures, subapically with transverse concavity. Postscutellum sharply, transversely convex, anterior portion transversely deeply concave. Lower half of mesopleuron with dense punctures (Fig. 5). Punctures in upper and anterior portion of mesopleuron correspondingly sparse. Speculum and its surrounding areas smooth and shiny. Upper end of epicnemial carina reaching about 0.6 distance to subalar prominence. Metapleuron convex, with dense, fine punctures. Juxtacoxal carina indistinct. Juxtacoxal area with dense oblique longitudinal wrinkles. Submetapleural carina complete. Wings brownish hyaline. Fore wing with vein 1cu-a slightly distal to 1-M. Areolet with short petiole, receiving vein 2m-cu at posterior 0.3. 2–Cu 2.0 times as long as 2cu-a. Hind wing vein 1-cu about 2.0 times as long as cu-a; cu-a slightly reclivous. Hind tibia 0.86 times as long as basal three segments of hind tarsus combined. Ratio of length of hind tarsomeres 1:2:3:4:5 is 2.7:1.8:1.3:0.8:1.1. Longest hind tibial spur (Fig. 7) slightly longer than widest width of hind tibia. Tarsal claws (Fig. 8) with 5–6 teeth at base. Propodeum (Fig. 6) with complete, strong median longitudinal, lateral longitudinal and pleural carinae. Lateral section of posterior transverse carina strong. Area superomedia combined with areas basalis and petiolaris. Costula absent. Basal-median portion between median longitudinal carinae with irregular, weak wrinkles. Area petiolaris almost smooth, lateral margins with weak transverse wrinkles. Between median longitudinal and lateral carinae smooth, with distinct punctures; between lateral longitudinal and pleural carinae with distinct punctures and long brown hairs. Propodeal spiracle almost circular.

**Metasoma.** First tergite about 1.9 times as long as apical width; median dorsal carinae reaching apical 0.2; interspace between median dorsal carinae slightly concave and almost smooth and shiny. Lateral parts of postpetiole with dense transverse wrinkles. Dorsolateral and ventrolateral carinae complete. Spiracle circular, small, evidently convex, located at middle of first tergite. Second tergite (Fig. 9) almost shiny, 1.27 times as long as basal width, 0.93 times as long as apical width; basal 0.35 with a pair of median longitudinal carinae; from spiracle to base with a strong carina; with shallow, sparse, uneven punctures. Third and following tergites slightly shagreened. Third tergite with shallow, small, indistinct punctures; 0.95 times as long as basal width, 1.03 times as long as apical width. Lateral margins of tergites 4 to 6 almost parallel. Tergite 7 transverse. Tergite 8 smooth, shiny, basal-median portion concave, apical portion weakly projecting upwardly. Sternites 4 to 6 strongly sclerotized.

**Color** (Fig. 1). Black, except the following. Flagellomeres 18 to 27 white. Lateral portions of clypeus, maxillary and labial palpi yellowish brown. Median portion of mandible reddish brown, basal blackish brown, teeth black. Upper-posterior corner of pronotum, tegula, small indistinct spot on subalar ridge, anterior and middle legs except coxae, apical apex of hind trochanter, extreme base of hind femur, hind tibia except apical portion brownish black and base slightly blackish, reddish brown. Apical portion of tergite 1, tergites 2 and 3, basal portion of tergite 4, lateral portions of tergites 5 and 6 brownish red. Pterostigma blackish brown. Veins brownish black.

#### Remarks.

This new species is similar to *Notopygus emarginatus* Holmgren, 1857, and *Notopygus eurus* Kasparyan, 2002, in having a white ring on the antenna, tergite 2 with a pair of median longitudinal carinae in basal portion, mandible partly brownish red, median tergites usually reddish brown, but can be distinguished from *Notopygus emarginatus* by the key mentioned above, and can be distinguished from *Notopygus eurus* by the following combination of characters. Frons strongly divided into two parts, lower part deeply concave, smooth; dorsal part flat, with indistinct, weak, irregular wrinkles. Scuto-scutellar groove with short longitudinal wrinkles. Fore wing with vein 1cu-a distal to 1/M. Hind wing vein 1-cu 2.0 times as long as cu-a. *Notopygus eurus*: Frons not separated into two parts. Scuto-scutellar groove smooth without longitudinal wrinkles. Fore wing with vein 1cu-a opposite 1/M. Hind wing vein 1-cu about as long as or slightly longer than cu-a.

### Notopygus emarginatus

Holmgren, 1857

http://species-id.net/wiki/Notopygus_emarginatus

**Figures 11.**
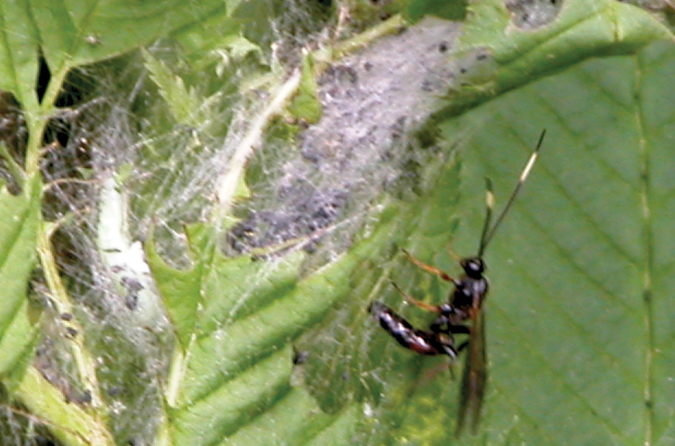
*Notopygus emarginatus* Holmgren, 1857. **11** Female ovipositing in larva of *Neurotoma sibirica* in the host’s web.

Figures 11 [Fig F3] [Fig F4] –17

Notopygus emarginatus Holmgren, 1857. Kongliga Svenska Vetenskapsakademiens Handlingar, 1(1)(1855): 115.
Notopygus insignis Kriechbaumer, 1891: Chen et al. 2007: Forest Pest and Disease, 26(6): 8.

#### Specimens examined.

8 females, CHINA: Haicheng, Liaoning Province, 5 to 30 June 2004, leg. Tian-Lin Chen. 1 female, CHINA: Benxi County, Liaoning Province, 15 June 2006, leg. Mao-Ling Sheng. 1 female, CHINA: Baishilazi Natural Reserve, Kuandian County, Liaoning Province, 7 July 2011, Intercept trap. 1 female, CHINA: Benxi County, Liaoning Province, 12 August 2013, leg. Mao-Ling Sheng.

**Figures 12. F3:**
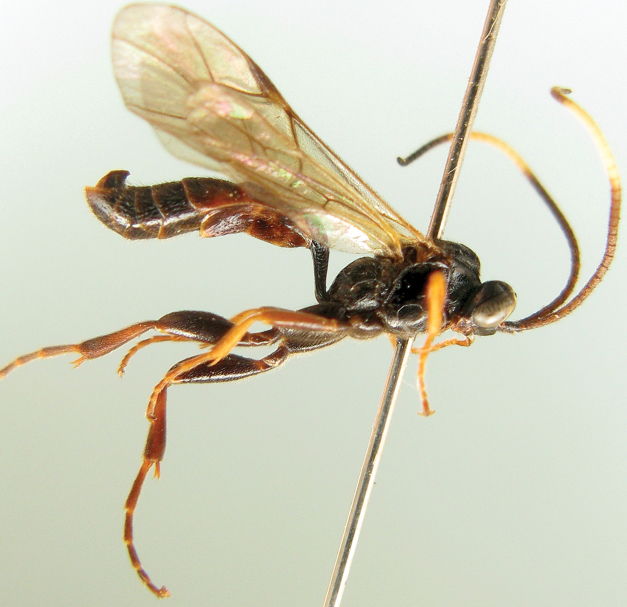
*Notopygus emarginatus* (GSFPM). **12** Female habitus, lateral view.

**Figures 13–15. F4:**
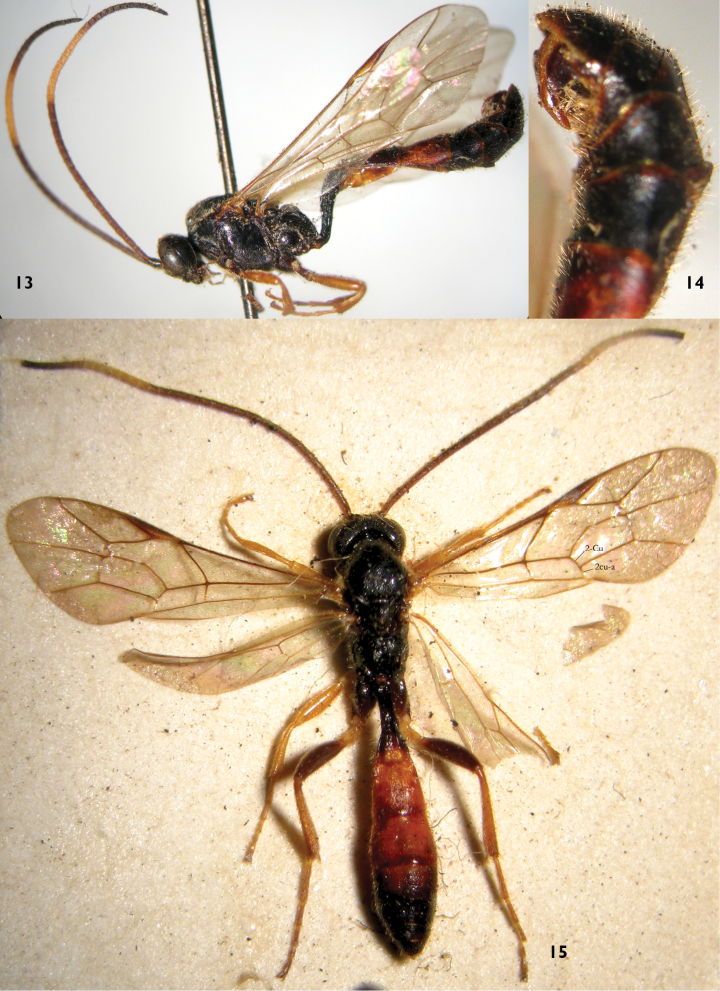
*Notopygus emarginatus* (NHM). **13–14** Female **13** Habitus, lateral view **14** Apical portion of metasoma, lateral view **15** Male habitus, dorsal view.

**Figures 16–17.**
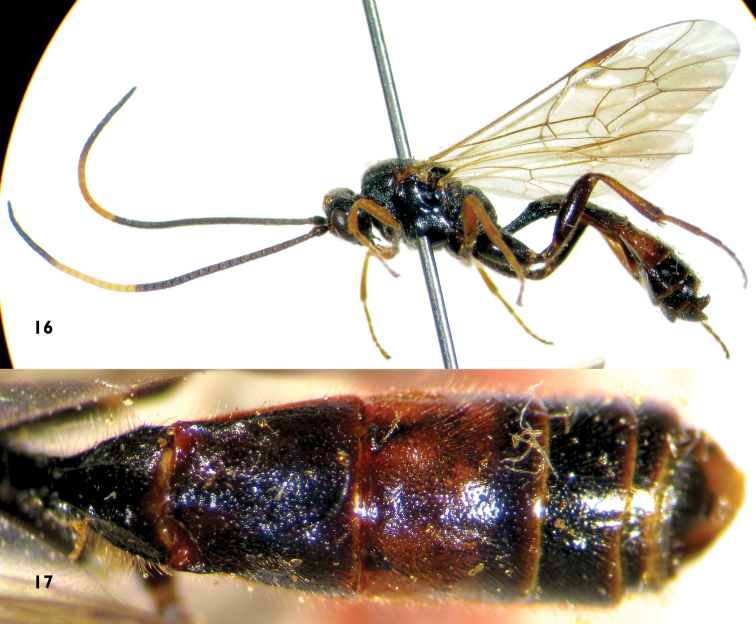
*Notopygus emarginatus* (ZSM). Female **16** Habitus, lateral view **17** Metasoma, dorsal view.

#### Hosts.

*Neurotoma sibirica* Gussakovskij (Hymenoptera, Pamphiliidae).

#### Host plants.

*Sorbaria sorbifolia* (L.); *Crataegus pinnatifida* Bunge.

Intraspecific variation. We also examined specimens deposited in the Natural History Museum, London (NHM) (Figs 13–15) and the Zoologische Staatssammlung München (ZSM) (Figs 16, 17). The female specimens have the same characters, except the color of the basal portion of the antenna and hind femora are a little variable. Tergites 2, 3 and the basal part of tergite 4 of males (Figure 15) are reddish brown.

### Notopygus raricolor

(Aubert, 1985)

Homaspis raricolor Aubert, 1985. Bulletin de la Société Entomologique de Mulhouse, 1985 (octobre-décembre): 49–58.

#### Remarks.

The species was redescribed and discussed by Kasparyan (2002). Specimens have not been examined. Known from Sichuan Province, China (Aubert 1985, Kasparyan 2002). Host is unknown.

## Supplementary Material

XML Treatment for Notopygus

XML Treatment for Notopygus longiventris

XML Treatment for Notopygus emarginatus

XML Treatment for Notopygus raricolor
